# Supplementary material for: A Postmortem MRI Study of Cerebrovascular Disease and Iron Content at End-Stage of Fragile X-Associated Tremor/Ataxia Syndrome
Source: Cells. 2023 Jul 20;12(14):1898. doi: 10.3390/cells12141898 (PMC10377990; doi:10.3390/cells12141898)
Supplement: Supplementary file 1 [file cells-12-01898-s001.zip › cells-2467263-supplementary.pdf]

Supplementary Materials

Table S1. Variances of the principal components (PC)

	PC1	PC2	PC3	PC4	PC5	PC6	PC7	PC8
**Standard deviation**	3.412	3.215	2.113	1.711	1.489	1.398	1.179	1.066
**Proportion of variance**	0.291	0.258	0.112	0.073	0.055	0.049	0.035	0.028
**Cumulative proportion**	0.291	0.550	0.661	0.734	0.790	0.839	0.873	0.902
	**PC9**	**PC10**	**PC11**	**PC12**	**PC13**	**PC14**	**PC15**	**PC16**
**Standard deviation**	0.999	0.937	0.828	0.717	0.652	0.538	0.376	0.000
**Proportion of variance**	0.025	0.022	0.017	0.013	0.011	0.007	0.004	0.000
**Cumulative proportion**	0.927	0.949	0.966	0.979	0.989	0.996	1.000	1.000

Table S2. Contributions of variables in the determination of PCs

Variables	Dim.1	Dim.2	Dim.3	Dim.4	Dim.5	Dim.6	Dim.7	Dim.8
Age	0.317	0.022	0.374	0.032	0.002	0.007	0.040	0.011
MCP	0.003	0.093	0.291	0.012	0.008	0.123	0.335	0.059
h.pallidus	0.026	0.002	0.722	0.026	0.000	0.000	0.032	0.054
h.genu	0.127	0.169	0.248	0.156	0.118	0.068	0.006	0.010
h.splenim	0.190	0.346	0.214	0.072	0.051	0.014	0.013	0.014
h.anterior	0.210	0.610	0.002	0.016	0.008	0.004	0.000	0.023
h.posterior	0.503	0.205	0.005	0.019	0.118	0.021	0.008	0.007
h.inferior	0.406	0.021	0.056	0.181	0.027	0.009	0.069	0.067
h.frontal	0.137	0.570	0.002	0.096	0.020	0.030	0.038	0.002
h.parietal	0.472	0.086	0.006	0.005	0.086	0.227	0.027	0.000
h.temporal	0.023	0.216	0.012	0.094	0.118	0.028	0.000	0.420
h.occipital	0.152	0.158	0.030	0.118	0.002	0.405	0.092	0.025
v.putamean	0.000	0.592	0.026	0.176	0.029	0.056	0.001	0.009
d.putamean	0.562	0.091	0.034	0.145	0.002	0.057	0.017	0.001
v.pallidus	0.003	0.822	0.054	0.012	0.021	0.028	0.013	0.021
d.pallidus	0.471	0.293	0.001	0.093	0.002	0.053	0.064	0.005
v.caudate	0.006	0.814	0.002	0.001	0.005	0.071	0.018	0.007
d.caudate	0.742	0.053	0.010	0.094	0.011	0.003	0.005	0.000
v.thalamus	0.000	0.764	0.115	0.019	0.000	0.061	0.004	0.005
d.thalamus	0.872	0.012	0.001	0.002	0.021	0.048	0.014	0.008
v.hippocampus	0.043	0.106	0.265	0.008	0.381	0.001	0.000	0.049
d.hippocampus	0.585	0.173	0.018	0.014	0.014	0.013	0.048	0.020
v.amygdala	0.324	0.002	0.101	0.012	0.388	0.072	0.053	0.022
d.amygdala	0.636	0.049	0.082	0.010	0.008	0.006	0.065	0.000
v.subthalamic n.	0.106	0.605	0.081	0.075	0.024	0.036	0.000	0.000
d.subthalamic n.	0.612	0.211	0.007	0.000	0.003	0.106	0.008	0.000
v.red n.	0.034	0.181	0.386	0.018	0.003	0.035	0.052	0.071
d.red n.	0.732	0.013	0.005	0.030	0.055	0.025	0.065	0.003
v.nigra	0.001	0.103	0.364	0.227	0.036	0.003	0.078	0.056

d.nigra	0.658	0.135	0.013	0.000	0.000	0.017	0.000	0.024
v.dentate n.	0.089	0.434	0.157	0.057	0.109	0.121	0.005	0.002
d.dentate n.	0.463	0.062	0.000	0.003	0.150	0.018	0.079	0.000
v.wm	0.014	0.826	0.002	0.024	0.002	0.007	0.036	0.000
d.wm	0.629	0.070	0.017	0.053	0.020	0.065	0.001	0.000
v.gm	0.000	0.430	0.271	0.124	0.023	0.009	0.005	0.073
v.cbwm	0.000	0.627	0.068	0.217	0.003	0.000	0.002	0.039
d.cbwm	0.522	0.060	0.075	0.076	0.134	0.027	0.000	0.019
v.cbgm	0.157	0.177	0.310	0.000	0.184	0.036	0.045	0.000
ICH	0.475	0.071	0.027	0.329	0.021	0.006	0.024	0.009
CMB	0.342	0.063	0.014	0.282	0.011	0.040	0.025	0.000
**Variables**	**Dim.9**	**Dim.10**	**Dim.11**	**Dim.12**	**Dim.13**	**Dim.14**	**Dim.15**	**Dim.16**
Age	0.003	0.017	0.076	0.075	0.000	0.024	0.000	0.000
MCP	0.007	0.001	0.033	0.001	0.000	0.035	0.000	0.000
h.pallidus	0.104	0.000	0.000	0.002	0.029	0.002	0.000	0.000
h.genu	0.002	0.002	0.017	0.026	0.033	0.003	0.013	0.000
h.splenim	0.009	0.040	0.027	0.001	0.009	0.000	0.001	0.000
h.anterior	0.055	0.000	0.005	0.004	0.044	0.012	0.006	0.000
h.posterior	0.070	0.004	0.003	0.009	0.010	0.011	0.007	0.000
h.inferior	0.006	0.076	0.034	0.023	0.016	0.009	0.001	0.000
h.frontal	0.078	0.000	0.005	0.005	0.014	0.001	0.004	0.000
h.parietal	0.000	0.001	0.046	0.026	0.002	0.009	0.008	0.000
h.temporal	0.011	0.014	0.021	0.027	0.015	0.002	0.000	0.000
h.occipital	0.006	0.003	0.002	0.004	0.000	0.000	0.003	0.000
v.putamean	0.055	0.011	0.015	0.010	0.005	0.013	0.001	0.000
d.putamean	0.001	0.073	0.003	0.004	0.006	0.000	0.007	0.000
v.pallidus	0.002	0.005	0.002	0.004	0.000	0.010	0.000	0.000
d.pallidus	0.000	0.008	0.005	0.000	0.001	0.001	0.000	0.000
v.caudate	0.037	0.000	0.027	0.006	0.001	0.005	0.000	0.000
d.caudate	0.030	0.003	0.012	0.033	0.001	0.003	0.000	0.000
v.thalamus	0.013	0.009	0.001	0.000	0.004	0.005	0.000	0.000
d.thalamus	0.001	0.003	0.017	0.001	0.001	0.001	0.000	0.000
v.hippocampus	0.118	0.001	0.001	0.002	0.001	0.005	0.018	0.000
d.hippocampus	0.084	0.018	0.000	0.000	0.007	0.004	0.002	0.000
v.amygdala	0.009	0.011	0.006	0.000	0.000	0.000	0.000	0.000
d.amygdala	0.037	0.048	0.001	0.023	0.006	0.027	0.003	0.000
v.subthalamic n.	0.005	0.024	0.000	0.008	0.014	0.013	0.007	0.000
d.subthalamic n.	0.010	0.005	0.010	0.011	0.014	0.003	0.001	0.000
v.red n.	0.035	0.104	0.056	0.001	0.024	0.001	0.000	0.000
d.red n.	0.002	0.022	0.000	0.001	0.046	0.000	0.000	0.000
v.nigra	0.000	0.069	0.000	0.050	0.007	0.005	0.001	0.000
d.nigra	0.011	0.017	0.061	0.001	0.003	0.023	0.037	0.000
v.dentate n.	0.000	0.003	0.010	0.000	0.007	0.000	0.008	0.000
d.dentate n.	0.000	0.165	0.000	0.058	0.000	0.001	0.001	0.000

v.wm	0.014	0.018	0.001	0.019	0.001	0.034	0.001	0.000
d.wm	0.018	0.000	0.108	0.001	0.010	0.005	0.002	0.000
v.gm	0.002	0.028	0.020	0.005	0.003	0.006	0.001	0.000
v.cbwm	0.022	0.007	0.005	0.000	0.003	0.007	0.000	0.000
d.cbwm	0.018	0.020	0.020	0.016	0.011	0.000	0.002	0.000
v.cbgm	0.010	0.010	0.014	0.019	0.033	0.004	0.000	0.000
ich	0.019	0.000	0.001	0.000	0.014	0.002	0.002	0.000
cmb	0.095	0.035	0.022	0.037	0.031	0.001	0.002	0.000

Abbreviations: d.~ = iron content; h.~ = T2 hyperintensities; v.~ = volume; cbwm = cerebellar white matter; cmb = cerebral microbleed; gm = gray matter; ich = intracranial hemorrhage; MCP = the middle cerebellar peduncle sign; n. = nucleus; wm = white matter

Table S3. The quality of representation of the variables on the PCs (cos2)

Variables	Dim.1	Dim.2	Dim.3	Dim.4	Dim.5	Dim.6	Dim.7	Dim.8
Age	0.317	0.022	0.374	0.032	0.002	0.007	0.040	0.011
MCP	0.003	0.093	0.291	0.012	0.008	0.123	0.335	0.059
h.pallidus	0.026	0.002	0.722	0.026	0.000	0.000	0.032	0.054
h.genu	0.127	0.169	0.248	0.156	0.118	0.068	0.006	0.010
h.splenim	0.190	0.346	0.214	0.072	0.051	0.014	0.013	0.014
h.anterior	0.210	0.610	0.002	0.016	0.008	0.004	0.000	0.023
h.posterior	0.503	0.205	0.005	0.019	0.118	0.021	0.008	0.007
h.inferior	0.406	0.021	0.056	0.181	0.027	0.009	0.069	0.067
h.frontal	0.137	0.570	0.002	0.096	0.020	0.030	0.038	0.002
h.parietal	0.472	0.086	0.006	0.005	0.086	0.227	0.027	0.000
h.temporal	0.023	0.216	0.012	0.094	0.118	0.028	0.000	0.420
h.occipital	0.152	0.158	0.030	0.118	0.002	0.405	0.092	0.025
v.putamean	0.000	0.592	0.026	0.176	0.029	0.056	0.001	0.009
d.putamean	0.562	0.091	0.034	0.145	0.002	0.057	0.017	0.001
v.pallidus	0.003	0.822	0.054	0.012	0.021	0.028	0.013	0.021
d.pallidus	0.471	0.293	0.001	0.093	0.002	0.053	0.064	0.005
v.caudate	0.006	0.814	0.002	0.001	0.005	0.071	0.018	0.007
d.caudate	0.742	0.053	0.010	0.094	0.011	0.003	0.005	0.000
v.thalamus	0.000	0.764	0.115	0.019	0.000	0.061	0.004	0.005
d.thalamus	0.872	0.012	0.001	0.002	0.021	0.048	0.014	0.008
v.hippocampus	0.043	0.106	0.265	0.008	0.381	0.001	0.000	0.049
d.hippocampus	0.585	0.173	0.018	0.014	0.014	0.013	0.048	0.020
v.amygdala	0.324	0.002	0.101	0.012	0.388	0.072	0.053	0.022
d.amygdala	0.636	0.049	0.082	0.010	0.008	0.006	0.065	0.000
v.subthalamic n.	0.106	0.605	0.081	0.075	0.024	0.036	0.000	0.000
d.subthalamic n.	0.612	0.211	0.007	0.000	0.003	0.106	0.008	0.000
v.red n.	0.034	0.181	0.386	0.018	0.003	0.035	0.052	0.071
d.red n.	0.732	0.013	0.005	0.030	0.055	0.025	0.065	0.003
v.nigra	0.001	0.103	0.364	0.227	0.036	0.003	0.078	0.056
d.nigra	0.658	0.135	0.013	0.000	0.000	0.017	0.000	0.024

v.dentate n.	0.089	0.434	0.157	0.057	0.109	0.121	0.005	0.002
d.dentate n.	0.463	0.062	0.000	0.003	0.150	0.018	0.079	0.000
v.WM	0.014	0.826	0.002	0.024	0.002	0.007	0.036	0.000
d.WM	0.629	0.070	0.017	0.053	0.020	0.065	0.001	0.000
v.GM	0.000	0.430	0.271	0.124	0.023	0.009	0.005	0.073
v.CBWM	0.000	0.627	0.068	0.217	0.003	0.000	0.002	0.039
d.CBWM	0.522	0.060	0.075	0.076	0.134	0.027	0.000	0.019
v.CBGM	0.157	0.177	0.310	0.000	0.184	0.036	0.045	0.000
ICH	0.475	0.071	0.027	0.329	0.021	0.006	0.024	0.009
CMB	0.342	0.063	0.014	0.282	0.011	0.040	0.025	0.000

**Variables**	**Dim.9**	**Dim.10**	**Dim.11**	**Dim.12**	**Dim.13**	**Dim.14**	**Dim.15**	**Dim.16**
Age	0.003	0.017	0.076	0.075	0.000	0.024	0.000	0.000
MCP	0.007	0.001	0.033	0.001	0.000	0.035	0.000	0.000
h.pallidus	0.104	0.000	0.000	0.002	0.029	0.002	0.000	0.000
h.genu	0.002	0.002	0.017	0.026	0.033	0.003	0.013	0.000
h.splenim	0.009	0.040	0.027	0.001	0.009	0.000	0.001	0.000
h.anterior	0.055	0.000	0.005	0.004	0.044	0.012	0.006	0.000
h.posterior	0.070	0.004	0.003	0.009	0.010	0.011	0.007	0.000
h.inferior	0.006	0.076	0.034	0.023	0.016	0.009	0.001	0.000
h.frontal	0.078	0.000	0.005	0.005	0.014	0.001	0.004	0.000
h.parietal	0.000	0.001	0.046	0.026	0.002	0.009	0.008	0.000
h.temporal	0.011	0.014	0.021	0.027	0.015	0.002	0.000	0.000
h.occipital	0.006	0.003	0.002	0.004	0.000	0.000	0.003	0.000
v.putamean	0.055	0.011	0.015	0.010	0.005	0.013	0.001	0.000
d.putamean	0.001	0.073	0.003	0.004	0.006	0.000	0.007	0.000
v.pallidus	0.002	0.005	0.002	0.004	0.000	0.010	0.000	0.000
d.pallidus	0.000	0.008	0.005	0.000	0.001	0.001	0.000	0.000
v.caudate	0.037	0.000	0.027	0.006	0.001	0.005	0.000	0.000
d.caudate	0.030	0.003	0.012	0.033	0.001	0.003	0.000	0.000
v.thalamus	0.013	0.009	0.001	0.000	0.004	0.005	0.000	0.000
d.thalamus	0.001	0.003	0.017	0.001	0.001	0.001	0.000	0.000
v.hippocampus	0.118	0.001	0.001	0.002	0.001	0.005	0.018	0.000
d.hippocampus	0.084	0.018	0.000	0.000	0.007	0.004	0.002	0.000
v.amygdala	0.009	0.011	0.006	0.000	0.000	0.000	0.000	0.000
d.amygdala	0.037	0.048	0.001	0.023	0.006	0.027	0.003	0.000
v.subthalamic n.	0.005	0.024	0.000	0.008	0.014	0.013	0.007	0.000
d.subthalamic n.	0.010	0.005	0.010	0.011	0.014	0.003	0.001	0.000
v.red n.	0.035	0.104	0.056	0.001	0.024	0.001	0.000	0.000
d.red n.	0.002	0.022	0.000	0.001	0.046	0.000	0.000	0.000
v.nigra	0.000	0.069	0.000	0.050	0.007	0.005	0.001	0.000
d.nigra	0.011	0.017	0.061	0.001	0.003	0.023	0.037	0.000
v.dentate n.	0.000	0.003	0.010	0.000	0.007	0.000	0.008	0.000
d.dentate n.	0.000	0.165	0.000	0.058	0.000	0.001	0.001	0.000
v.wm	0.014	0.018	0.001	0.019	0.001	0.034	0.001	0.000

d.wm	0.018	0.000	0.108	0.001	0.010	0.005	0.002	0.000
v.gm	0.002	0.028	0.020	0.005	0.003	0.006	0.001	0.000
v.cbwm	0.022	0.007	0.005	0.000	0.003	0.007	0.000	0.000
d.cbwm	0.018	0.020	0.020	0.016	0.011	0.000	0.002	0.000
v.cbgm	0.010	0.010	0.014	0.019	0.033	0.004	0.000	0.000
ich	0.019	0.000	0.001	0.000	0.014	0.002	0.002	0.000
cmb	0.095	0.035	0.022	0.037	0.031	0.001	0.002	0.000

Abbreviations: d.~ = iron content; h.~ = T2 hyperintensities; v.~ = volume; cbwm = cerebellar white matter; cmb = cerebral microbleed; gm = gray matter; ich = intracranial hemorrhage; MCP = the middle cerebellar peduncle sign; n. = nucleus; wm = white matter

Table S4. The quality of representation of the individuals on the PCs (cos2)

Individuals	Dim.1	Dim.2	Dim.3	Dim.4	Dim.5	Dim.6	Dim.7	Dim.8
**B001**	0.056	0.106	0.275	0.015	0.074	0.113	0.048	0.039
**B002**	0.638	0.075	0.154	0.084	0.000	0.009	0.007	0.002
**B003**	0.796	0.007	0.038	0.028	0.037	0.000	0.000	0.012
**B004**	0.400	0.465	0.001	0.043	0.001	0.011	0.037	0.019
**B005**	0.375	0.138	0.001	0.335	0.003	0.111	0.000	0.021
**B008**	0.030	0.121	0.438	0.033	0.054	0.114	0.108	0.027
**B009**	0.312	0.482	0.049	0.000	0.005	0.078	0.058	0.009
**B014**	0.007	0.235	0.000	0.063	0.102	0.104	0.239	0.039
**B015**	0.039	0.312	0.001	0.025	0.045	0.012	0.010	0.243
**B017**	0.019	0.399	0.304	0.094	0.051	0.036	0.000	0.017
**B018**	0.173	0.411	0.085	0.003	0.127	0.086	0.009	0.000
**B019**	0.364	0.133	0.042	0.180	0.014	0.018	0.002	0.126
**B020**	0.073	0.004	0.568	0.016	0.059	0.008	0.070	0.017
**B023**	0.151	0.149	0.042	0.127	0.001	0.025	0.006	0.006
**B025**	0.001	0.333	0.011	0.000	0.465	0.053	0.032	0.011
**B026**	0.133	0.337	0.111	0.082	0.023	0.010	0.035	0.006
**Individuals**	Dim.9	Dim.10	Dim.11	Dim.12	Dim.13	Dim.14	Dim.15	Dim.16
**B001**	0.184	0.033	0.004	0.030	0.001	0.019	0.003	0.000
**B002**	0.006	0.000	0.000	0.009	0.000	0.000	0.016	0.000
**B003**	0.011	0.044	0.012	0.000	0.000	0.003	0.011	0.000
**B004**	0.000	0.000	0.012	0.000	0.007	0.002	0.000	0.000
**B005**	0.003	0.000	0.006	0.003	0.003	0.000	0.000	0.000
**B008**	0.044	0.001	0.000	0.024	0.002	0.004	0.000	0.000
**B009**	0.001	0.000	0.000	0.002	0.002	0.001	0.000	0.000
**B014**	0.070	0.010	0.042	0.044	0.029	0.017	0.000	0.000
**B015**	0.019	0.193	0.035	0.032	0.019	0.017	0.000	0.000
**B017**	0.000	0.040	0.012	0.010	0.006	0.011	0.001	0.000
**B018**	0.007	0.058	0.006	0.002	0.017	0.010	0.004	0.000
**B019**	0.021	0.065	0.012	0.006	0.017	0.000	0.001	0.000
**B020**	0.047	0.002	0.028	0.025	0.004	0.079	0.001	0.000

**B023**	0.060	0.001	0.326	0.005	0.094	0.004	0.005	0.000
**B025**	0.071	0.003	0.000	0.005	0.015	0.000	0.000	0.000
**B026**	0.060	0.003	0.007	0.125	0.050	0.004	0.016	0.000
